# E-Textiles for Healthy Ageing

**DOI:** 10.3390/s19204463

**Published:** 2019-10-15

**Authors:** Kai Yang, Beckie Isaia, Laura J.E. Brown, Steve Beeby

**Affiliations:** 1 Electronics and Computer Science, University of Southampton, Southampton SO17 1BJ, UK; ky2@ecs.soton.ac.uk (K.Y.); R.J.Isaia@soton.ac.uk (B.I.); 2 School of Health Sciences, University of Manchester, Manchester M13 9PL, UK; laura.brown@manchester.ac.uk

**Keywords:** healthy ageing, e-textiles, wearable devices, disease and disability, sensors, actuators, rehabilitation

## Abstract

The ageing population has grown quickly in the last half century with increased longevity and declining birth rate. This presents challenges to health services and the wider society. This review paper considers different aspects (e.g., physical, mental, and social well-being) of healthy ageing and how health devices can help people to monitor health conditions, treat diseases and promote social interactions. Existing technologies for addressing non-physical (e.g., Alzheimer’s, loneliness) and physical (e.g., stroke, bedsores, and fall) related challenges are presented together with the drivers and constraints of using e-textiles for these applications. E-textiles provide a platform that enables unobtrusive and ubiquitous deployment of sensors and actuators for healthy ageing applications. However, constraints remain on battery, integration, data accuracy, manufacturing, durability, ethics/privacy issues, and regulations. These challenges can only effectively be met by interdisciplinary teams sharing expertise and methods, and involving end users and other key stakeholders at an early stage in the research.

## 1. Introduction

The proportion of the world’s population over 60 years old is estimated to increase from 12% in 2015 to 22% by 2050 [1]. Achieving healthy ageing will improve each person’s feeling of wellbeing, reduce healthcare costs and increase the ability of individuals to work and enjoy life. Healthy ageing is much more than just physical wellbeing, important though that is. In addition to common physical symptoms and conditions that are associated with ageing, technology can also assist in improving cognitive ability, social interaction and mood, prolonging independence and promoting healthy behaviour. This review reflects on the definition of healthy ageing, the role of sensors and personal health devices (PHDs) and, in particular, focuses on how electronic textiles (e-textiles or smart fabrics) incorporating sensing functionality can assist in achieving healthy ageing. The E-textiles technologies presented in this paper are also relevant for supporting healthy lifestyles in general.

There are many examples of PHDs being used to monitor physical and mental wellbeing with the associated benefits of encouraging activities and maintaining functions. For example, fitness trackers with sensors embedded for monitoring daily activities (e.g., steps, distance, calories burned) [2] and heart rate [3]; adhesive patches for monitoring glucose level, heart rate and stress [4]; wearable electrodes for biopotential monitoring such as electrocardiogram (ECG), electromyogram (EMG) and electroencephalogram (EEG) [5]. PHDs exist in different forms, such as wristbands, gel patches as well as integrated into clothing.

E-textiles are advanced textiles that include electronic functionality ranging from conductive tracks to sensing/actuating, communications and microprocessing. The market for e-textiles is projected to reach $2 billion by 2029 [6]. The motivation for incorporating PHDs and electronic functionality into textiles in order to assist health ageing is clear. Textiles provide a comfortable ubiquitous platform that individuals are entirely familiar and comfortable with. The ability to hide the technology in garments can improve performance, remove stigma and improve compliance with technology amongst the ageing population.

Many age-related conditions can be monitored and treated with e-textile technology. For example, ECG T-shirts for early warning on cardiovascular disease [7], electrode garments for stroke rehabilitation [8] and incontinence treatment, insoles or shoes with embedded sensors for fall detection [9]. However, it is not straightforward to include electronic and sensing capability in textiles, and most of the existing e-textile technology does not deliver practical solutions with the required levels of functionality.

This review was associated with the output of the “Healthy Ageing” workshop organized by the E-textiles Network [10], which is an Engineering and Physical Sciences Research Council (EPSRC)- funded network to bring together researchers and developers from academia and industry interested in adding electronic functionality to textiles and their related products. The review has been augmented by a comprehensive evaluation of the capability of existing technologies and approaches to address a wide range of age-related challenges. A literature review was done based upon key words identified during the workshop for each of the physical and non-physical challenges identified and the corresponding e-textile (smart fabric) technologies. The review focused primarily on post-2015 publications and used Google Scholar and Web of Science. Given the breadth of topics covered in the review and the correspondingly overwhelming number of publications that could be cited, key exemplars from the literature were selected. Section 2 reviews what is healthy ageing and how inventions and technologies can support heathy ageing. Section 3 reviews the PHDs suitable for both non-physical and physical age related diseases. The drivers and constraints of using e-textiles in wearable technologies are also discussed. Section 4 reviews the e-textile applications on healthy ageing and the state-of-the-art technologies. The research and development required to realize the practical e-textile solutions that assist individuals to age more healthily are highlighted.

## 2. Healthy Ageing

The worldwide population aged 60 and above is expected to grow from 962 million in 2017 to 2.1 billion by 2050 [11]. This will place additional strain on the health services and there is a clear motivation to help people to stay active and productive for longer as they age. For example, the UK government’s mission is “to ensure people can enjoy at least five extra healthy, independent years of life by 2035, while narrowing the gap between the experiences of the richest and poorest” [12]. But what is meant by the term healthy ageing and what factors should be considered? The World Health Organization (WHO) define health as “a state of complete physical, mental and social well-being and not merely the absence of disease or infirmity”, indicating that there is more to healthy ageing than maintaining physical health. Friedman et al. [13] have also argued that definitions of healthy ageing may vary according to the specific goal that is trying to be achieved. For instance, different factors may be important if the goal is to minimize the costs of healthcare as opposed to enabling people to feel good for as long as possible.

**Figure 1:**
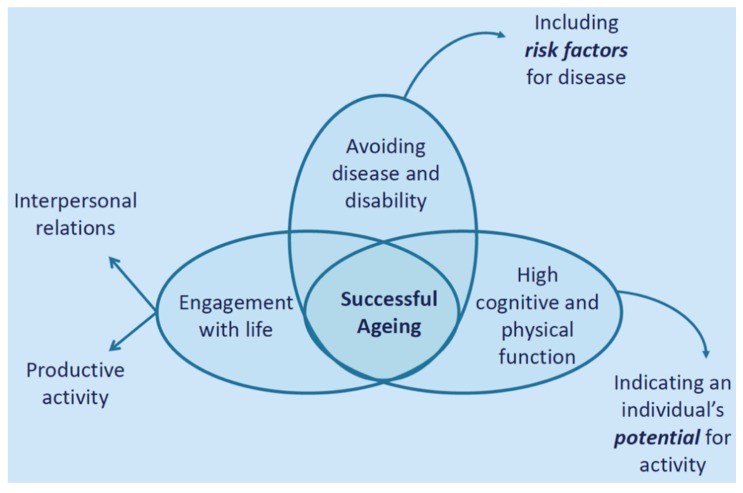
Rowe and Khan’s definition of successful ageing.

A commonly-used model of ‘successful ageing’ [14] discusses three key conditions that need to be met: (1) the avoidance of disease and disability, (2) the maintenance of high cognitive and physical function, and (3) continued engagement with life (Figure 1). Each of these domains is considered in more detail below.

### 2.1. Avoiding Disease and Disability

A recent study by Lu et al. [15] reviewed the measures of healthy ageing used in epidemiological studies. The most commonly measured aspects of metabolic and physical health were the self-reported presence of chronic diseases, such as cancer, lung or heart disease, diabetes or stroke. Other studies measured self-reported hypertension, as well as cardiovascular risk factors, biomarkers of kidney and cardiovascular function, Body Mass Index (BMI), pain, vision, audition, and sleep.

Age UK has published an almanac of disease profiles in later life presenting a reference on the frequency of major diseases, conditions and syndromes affecting older people in England [16]. It listed the most commonly-studied conditions and syndromes in older people as being:

- Cardiovascular diseases (e.g., hypertension, atrial fibrillation, coronary heart disease, heart failure and stroke)
- Neuropsychiatric conditions (e.g., dementia, depression, epilepsy, mental health)
- Respiratory (e.g., asthma, chronic obstructive, pulmonary disease)
- Endocrine (e.g., diabetes, hypothyroidism)
- Chronic kidney disease (stage 3 to 5)
- Cancer in the previous 5 year (excluding non-melanoma skin cancer)
- Additional common conditions (e.g., anaemia, osteoarthritis, osteoporosis)
- Additional syndromes (e.g., falls, fragility issues, incontinence, skin ulcers/pressure sores)

### 2.2. High Cognitive and Physical Function

In a consensus review of biomarkers of a healthy ageing phenotype, Lara et al. [17] listed the following elements of cognitive and physical function as important:

- Episodic memory
- Cognitive processing speed
- Executive functions
- Grip strength
- Gait speed
- Standing balance test etc.

These can also be related to functional ability to perform activities associated with daily living, such as walking, bathing or using a telephone [18].

### 2.3. Engagement with Life

Rowe and Khan break this down two sub-categories: (1) interpersonal relations and (2) productive activity. The former could be operationalized as levels of loneliness and perceived emotional and social support, whilst the later may relate an individual’s contribution to society, or their purpose in life, as defined by Ryff and Keyes [19].

### 2.4. Lay Perspectives

It is important to recognize that conceptualizations of healthy, or successful, ageing defined by clinicians and researchers may differ to those of lay older people. For example, Strawbridge et al. [20] studied 867 adults aged 65–99 years, of whom 18.8% were rated as ageing successfully by Rowe and Khan’s criteria, but 50.3% rated themselves as ageing successfully. Lay definitions of healthy or successful ageing tend to be broader and more multi-faceted, and includes an increased emphasis on psychosocial factors, such as attitude and acceptance [21]. There are also cultural differences in how aspects of healthy ageing are conceptualized and valued [22].

### 2.5. Interventions and Technologies to Support Healthy Ageing

It is clear that healthy ageing concerns more than just physical wellbeing. Given the very broad range of factors that may contribute to healthy ageing, there is a correspondingly broad range of objectives by which interventions and technologies can support healthy ageing. These include measures to:


Avoid disease & disabilityReduce risk factorsMaintain cognitive abilityIncrease functional abilityPromote independence

Reduce isolationPromote acceptance of age-related changeIncrease healthy behaviourPromote positive mood and attitude


## 3. Wearable Devices in General and the Need for E-Textiles

### 3.1. Wearable Technologies

**Figure 2:**
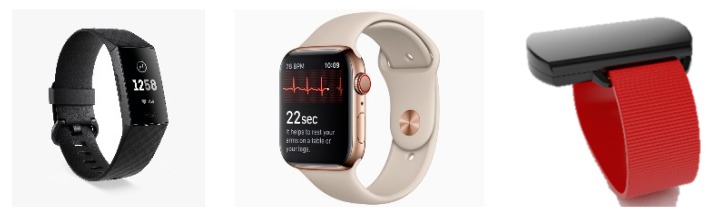
Fitbit health and fitness tracker (left), Apple watch (middle), SPHERE activity sensor (right) [24].

Personal health devices (PHDs) are defined as devices equipped with one or more sensors for monitoring physiological signals or activity levels. These are typically autonomous wireless devices and are therefore constrained in terms of the energy and processing power available, and they are typically, but not exclusively, wearable devices. Several examples of wearable devices exist that use conventional commercial off the shelf electronic and sensor components applied as, for example, wrist or lanyard-based systems as shown in Figure 2. The EPSRC IRC project SPHERE [23] developed a wrist mounted 6 axis inertial measurement unit for activity sensing [24]. Multiple inertial sensors distributed around an individual improve the accuracy of activity classification but require time synchronisation and orientation calibration. Wearable inertial measurement units have also been used to quantify and monitor axial bradykinesia (a feature of advanced Parkinson’s disease) while the subject performs routine domestic activities [25]. Such sensors can be wirelessly connected forming a Wireless Body Area Network (WBAN) or Body Sensor Network (BSN) typically using Zigbee or Bluetooth Low Energy (LE) communications protocols and can be readily incorporated into part of a larger smart home environment. Other examples of PHDs include pulse oximeters, blood pressure monitors, weighing scales, blood glucose meters, thermometers and fall detection sensors. Such sensors are an integral part of ambient assisted living systems and are an important source of data for health informatics.

### 3.2. Current and Future Applications of Devices to Support Healthy Ageing

The review of wearable PHDs for supporting healthy ageing and healthy lifestyles in general has been divided into two sections, with Section 3.2.1 covering non-physical (e.g., cognitive) health related challenges. Section 3.2.2 covers physical health and functional ability related challenges. Examples of existing technologies or underpinning research are given where possible and, in each case, the potential motivation for an e-textile based solution is discussed alongside the constraints imposed by such an e-textile version.

#### 3.2.1. Non-Physical Challenges Associated with Healthy Ageing

Age related mental illnesses (e.g., dementia, depression, loneliness) affects 15% of people aged 60 and over. Various technologies have demonstrated the promising potential benefits for addressing non-physical challenges associated with healthy ageing.

Dementia refers to a set of symptoms including memory loss, difficulties with thinking, problem solving and carrying out daily activities. It is caused by the decline in brain function and affects 50 million people worldwide [26]. Example technologies that can assist the daily living of people with memory loss include GPS and inertial tracking devices for location monitoring [27], wearable cameras [28] for recalling personal memory, and apps for promoting communicative actions [29]. Cognitive training apps [30] can help with time keeping and reminders. The use of ambient and wearable sensors and cameras has been studied by Stavropoulos et al. in an ambient assisted living framework (DemaWare 2) for the care of dementia patients [31]. The combination of sensor modalities yielded valuable results for activity detection, accurate sensor fusion and had clinical value in care. E-textile technology can be used to incorporate a range of GPS and inertial sensors in garments for tracking purposes and this offers the benefits of improving convenience for the user whilst simultaneously for providing data for activity classification. Similarly, tactile actuators and integrated textile speakers could also be used to provide prompts that aid memory recall. Textile implementations are constrained by the challenges of scaling manufacture of personalized solutions. There are technical challenges around textile actuators and speakers given the highly compliant nature of a typical fabric. To provide maximum benefit, the e-textile solution needs to integrate into a larger system as demonstrated by the DemaWare 2 framework.

Compliance with prescriptions is a big challenge for some people with age related mental illness. Example technologies that can support patient compliance include smartphone-based medication adherence applications providing scheduled reminders [32]. Other approaches aim to detect when medicines have been taken by, for example, incorporating sensors into medicine packaging [33] and activity recognition techniques detecting the motion associated with the twisting of a cap and the hand to mouth movement [34]. Inertial based sensors in clothing can also be used to detect the act of taking medicines and textile-based actuators/sounders can be used to provide a prompt for users to follow the prescription. Textiles can also provide a novel platform for drug delivery, for example by achieving the controlled release of drugs into garments or dressings [35]. The effectiveness compared to the oral medication would need to be investigated and challenges remain on implementing personalised individual solutions for each prescription.

Improving emotions and mood/emotion detection can enhance the wellbeing and provide early warning for mental illness. Information and communications technology (ICT) provides opportunities for improving moods through facilitating interactions (e.g., email, chat, friends’ forums, shared photos) and the wider provision of entertainment (e.g., videos and music) [36] and communication technologies (e.g., voice assistants). Exercise has been proved as an effective method to reduce the risk of depression [37] and technology can help incentivize individuals to become more active [38]. Emotion or mood can be detected using EEG for monitoring brain signals and through facial recognition [39]. Wearable e-textiles can be used to implement aspects of these technologies. For example, wearable comfortable EEG can be achieved by embedding EEG electrodes in a hat/headband in an unobtrusive way that also enhances the usability. However, constraints remain on the processing power available within wearable component which therefore needs to interface to smartphone. The accuracy and repeatability of EEG readings and the influence of electrodes position and motion artefacts needs to be considered. Textiles in general can be physically comforting and this can be further enhanced through the provision of haptic feedback (e.g., hug shirt [40]) or incorporating music playback [41] to enhance the interaction.

Sleeping quality is an important factor associated with mental health. Different technologies have been developed to monitor the sleep quality such as activity monitors, smartphone apps, baby monitors, embedded sensors in mattresses [42]. Heart rate and respiration sensor have also been used for sleep analysis [43]. E-textiles would provide a convenient, comfortable platform for hidden wearable sensors and facilitate combination of multiple sensors e.g., breathing, ECG, movement. Textile based sensors could also be integrated into bed linen. However, sufficient processing power is required for data analysis and pattern recognition.

Engagement with household activities (e.g., cooking, clothes washing, vacuuming) is another indicator of healthy ageing as some of these tasks can become difficult to perform. This can be measured by wearable inertial sensors for activity monitoring and classification [24,44], and location tracking [45]. Sensors on power points for appliances [46,47] and in furniture/carpet [48] can also be used to monitor how frequent household appliances are used. E-textiles would provide a convenient, comfortable platform for inertial and location sensors embedded in clothing. Pressure sensors can be integrated into furniture textiles/carpets to detect certain activities. Once again, the processing power required for accurate classification of tasks is challenging to incorporate into the textile.

Physical isolation/loneliness can lead to mental illness such as depression. Wearable sensors have been used for location sensing and for the detection of interaction with others e.g., through the monitoring of speech [49]. Invisible, comfortable and ubiquitous sensors can be integrated into textiles for tracking and location sensing as can breathing sensors or microphone for detecting speech. However, wireless communication/signal processing when outside the home could be challenging and complex electronics are required to identify speech accurately.

#### 3.2.2. Physical Health and Functional Ability Related Challenges Associated with Healthy Ageing

Ageing related disease, such as stroke and multiple sclerosis, has significant impact on the mobility of individuals. For example, over 70% of stroke survivors have reduced limb functions. Technology can assist in rehabilitation through assisted physiotherapy, functional electrical stimulation (FES) [50], sensors and robotics functionality [51]. Electrodes for FES can be located on textiles for wearable comfortable solutions leading to improved compliance with exercises and rehabilitation. Integrated inertial sensors in textiles/clothing can monitor exercise progress and be linked to gaming to motivate the intensive exercise. Soft robotics can potentially be implemented in textiles to assist mobility through the garment. However, individuality (the requirement for personalised solutions) is still an issue to achieve accurate movement especially for upper limb rehabilitation which involves many muscle groups. Assisting mobility using soft robotics requires high levels of actuation and implementations typically require tight fitting clothes.

Poor blood circulation and lack of muscle exercise can lead to many diseases such as deep vein thrombosis (DVT) and muscle atrophy. Assistive technologies for muscle exercise can improve both muscle functions and blood circulation. For example, electrical stimulation has been used to prevent muscle wasting [52]. Encouraging exercise through sensors, tracking and gamification [53,54] has been studied. Resistance training followed by heating [55] and the use of compression garments [56] can improve performance and reduce recovery times. Textiles provide a comfortable platform for wearable textile electrodes for FES, heart rate monitoring and pulse oximetry. Inertial sensors integrated in textiles and clothing can monitor activity and technique to improve function and enable gamification. Heating functionality built can be into clothing targeting specific locations to increase circulation and aid muscle growth. Accurate positioning of electrodes and blood flow sensors are necessary to maximise performance.

Urinary incontinence can happen at any age but it is more common in older adults due to a decline in how nerves and muscles signal the bladder and the loss of bladder elasticity. It affects 20% people over the age of 40 and 50% of the elderly in the nursing home. Disposable sensors has been used in assessment applications (identifying care plans) [57]. Implantable bladder sensors (pressure) and electrical stimulation have been used to manage the overactive bladder [58] and restore pelvic floor muscles [59]. Textile based moisture activated batteries or sensors can enable underwear to sense incontinence and provide an alert. More comfortable external textile-based electrodes can be used for electrical stimulation to exercise muscles and improve control although electrical stimulation will require tight fitting garment.

Cardiovascular disease is most common in people aged over 50 and the risk increases with age. Cardio health can be monitored using wearable health devices with heart rate sensors (e.g., smartwatches, chest straps) [60] although more useful diagnostic data (ECG waveform) typically requires more electrodes. Textile ECG electrodes on garments provides unobtrusive and comfortable interface and it is convenient (e.g., quick to put on) to use. Positioning and number of electrodes on a garment, along with garment design and fit requires further investigation to ensure quality of data sufficient to detect ECG waveform.

Bedsores are injuries to the skin and underlying tissue caused by prolonged pressure on the skin. It usually affected people with low mobility who spend more time in bed or chair/sofa for long periods time, in particular, for the elderly with vulnerable skins. Specialised wound dressing (e.g., containing silver, negative pressure wound therapy) [61] have been used in wound care. Pressure modulating mattresses/beds/overlays has been used to relieve pressure [62]. Pressure sensing arrays has been integrated on support surfaces for real time pressure measurement which could be a useful tool to help care providers to reposition patients to minimize the pressure ulcers [63]. Smart bandages with antibacterial properties (UV germicidal irradiation), and pressure and temperature monitoring capabilities can be used for wound dressing. Pressure sensing sheets can be integrated into clothing or mattress to monitor the pressure. There are cost constraints for disposable bandages and reusable germicidal bandages what can withstand cleaning cycles have potential to reduce the cost. Sensors embedded in bedding undergoes harsh washing cycles therefore durability needed to be considered.

Monitoring and managing glucose level is essential to diabetic patients. There are different types of measurement methods including the analysis of blood using the finger-prick test, flash glucose sensor (wirelessly addressable sensor placed on skin surface) [64] and continuous glucose monitoring (battery powered wireless system with implanted sensor) [65]. It can also be monitored through sweat [66] and tears [67]. Wearable, comfortable and less unsightly remote monitors (e.g., Senseonics Eversense) has been used for implanted continuous glucose sensor. Textiles provide convenient platform for sweat based glucose sensor. However, good alignment of the transmitter over skin mounted and implanted glucose sensors is required to get accurate measurement. Textile based glucose sensors are in their infancy.

Fall detection and early warning of falls (fall prediction) is useful to reduce the injuries caused by falling. An individual’s balance can be monitored through gait [68], inertial sensors [69] and foot-based sensors [70]. Predicting falls can be achieved by observing changes in time in data collected visually (e.g., Kinect systems) [71], from inertial [72] and floor-based sensors [73]. There are many opportunities for textile sensors including clothing based inertial sensors that are invisible to the wearer and easy to use. Balance monitoring can be achieved through pressure sensors incorporated in shoe insoles, socks, or in soft floor coverings such as carpets. Accurate textile pressure sensors are required for practical use and low-cost carpet based distributed sensor arrays is in infancy.

Monitoring respiratory pattern is relevant to the assessment of health state and can encourage breathing exercise. Controlled slow breathing and breathing techniques can affect a range of physiological parameters (e.g., heart rate, blood flow dynamics) that are associated with health and longevity [74]. Clothing based sensors can conveniently monitor breathing rate and type of breathing (e.g., shallow vs. deep). Smart textiles have been used for respiratory monitoring and thoraco-abdominal motion pattern evaluation [75]. However, the optimum sensing approach for textile-based solutions and the influence of garment type on sensor performance are not well understood.

### 3.3. Wearable Technology: The Motivation for E-Textiles

Textile implementations of sensors, electrodes and soft robotics have a large range of potential applications in supporting healthy ageing. A single development such as textile based inertial sensors can benefit a range of physical and non-physical challenges including the sensing of motion for the identification of household activities and taking of medicines; the provision of feedback during exercise or rehabilitation; fall prevention; and detection and encouragement of exercise through activity monitoring and gamification. The importance of encouraging physical activity in achieving healthy ageing should not be underestimated. Exercise maintains physical and cognitive function and reduces the risk of many of the factors identified above. For example, remaining physically active helps maintain cardio health, reduce the risk of fall, improve mood and reduce the risk of depression and dementia [76].

Similarly, textile-based electrodes also address a number of the societal and physical challenges identified above. This includes EEG monitoring for mood detection, ECG electrodes for monitoring exercise, cardio health and sleep, and FES electrodes for assisting rehabilitation and strengthening muscles to reduce muscle atrophy and prevent incontinence.

The benefits offered by e-textile implementation depend upon the application and the solution but there are a number of common advantages:

- Textile/clothing-based solutions provide a comfortable and familiar platform to users.
- E-textiles would enable unobtrusive and ubiquitous deployment sensors and actuators in clothing and furnishings.
- The integration in clothing will improve compliance (users might forget to use the conventional technology, but they always remember to get dressed).
- The unobtrusive nature of the technology will avoid any perceived stigma associated with wearing devices.
- Multiple sensors can be incorporated into a single platform (e.g., item of clothing) rather than requiring users to wear a number of separate devices.
- Information or alerts can be provided through the textile providing real time feedback to the user in a single platform.
- Textiles are the most common material that people interact with through, for example, clothing, soft toys, home furnishings and bed linen, and therefore providing an attractive platform for a range of applications.
- Ease of use and increased compliance can provide more data to better inform preventative interventions [77].

Certain constraints apply to e-textile implementations, and some of these are universal and apply to all the applications identified in Section 3.2.1 and Section 3.2.2. For example, e-textiles are reliant on conventional primary or secondary batteries and these are at present bulky, rigid and incompatible with the feel of the textile. For long-term monitoring applications, batteries require user intervention to replace or recharge and limit the level of integration since they must be removed prior to washing. Embedding electronic functionality can be achieved by coating a textile with smart or functional material [8], or embedding miniature MEMS sensors in the yarns [78] or fabric [79]. These approaches are not straightforward given the structure of the fabric, its fibrous nature and lack of consistency even along the same textile roll. The use of a textile substrate for coating-based approaches places constraints on the processing of the active film (e.g., processing temperature) which can limit the functionality of the active materials. Sensors must be reliably connected to signal processing electronics and power sources and embedding these technologies in an unobtrusive manner is not straightforward. The influence of the textile or clothing on the accuracy of the sensor data is unknown and will require calibration e.g., the data gathered from inertial sensors on garments will depend upon how tight fitting the clothing is. Electronic and sensing technologies incorporated during the manufacture of the textile must survive the associated manufacturing process (e.g., weaving, knitting, surface finishing) and e-textile processes must be compatible with mass manufacture. During use, textiles routinely experience harsh physical conditions (e.g., physical wear, bending and flexing, exposure to liquids) and ensuring solutions are robust and reliable is a significant challenge. E-textiles-based applications must consider safety considerations and the implications of data management and associated ethics/privacy issues must also be addressed. Ultimately, some e-textile applications may need to meet regulations that apply to medical devices.

Addressing these will enable e-textile technology to provide a user-friendly acceptable platform for assisting healthy ageing that address many of the limitations of existing conventional solutions [80]. The following section focuses on these constraints in more detail and identifies the specific research challenges that remain.

## 4. E-Textile Technologies for Assisting Healthy Ageing

As discussed above, E-textile technology can be used in many healthy ageing related applications. The technologies listed below address the e-textile solutions identified in Section 3.2.1 and Section 3.2.2. It also includes examples of the current state of the art and the research and development required to reach higher technology readiness levels (TRL) [81] and achieve a practically viable solution.

### 4.1. Inertial Sensors (e.g., Accelerometers)

These are used in inertial tracking, activity monitoring, monitoring physiotherapy exercises, gamification to encourage physical activity, fall prevention/detection, monitoring sleep quality, and compliance with prescriptions. Existing textile-based activity monitoring uses a variety of strain sensing techniques (see below) rather than inertial sensors [82]. Accelerometers can be mounted on to a textile using the Lilypad platform (sewn on rigid PCBs) [83]. Textile goniometers and accelerometers have been used to measure the flexion-extension angle of the knee in different motion tasks [84]. Improved integration in the fabric itself has been achieved in the EPSRC funded FETT project [85]. Research required includes achieving full inertial measurement by incorporating magnetometers and gyroscopes, understanding how the textile embodiment effects performance and the impact this has on activity classification. For example, the impact of mounting inertial sensors in loose clothing is not understood.

### 4.2. Fabric Electrodes

**Figure 3:**
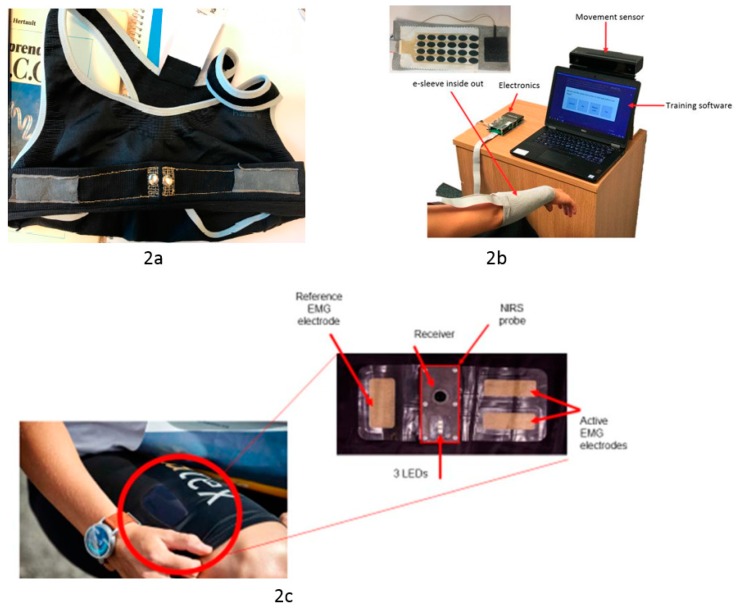
2a: ECG electrode sewn in bra [86], 2b: FES electrode embedded in sleeve [8], 2c: EMG electrode integrated in clothing [88].

These are used for biosignal sensing and stimulation. For example, ECG for heart rate for activity tracking, cardio health monitoring and sleep analysis. They are also used in EMG measurement to track muscle activity during physiotherapy, and in EEG for brain signal monitoring for mood detection. FES is used in assistive physiotherapy for rehabilitation and improved muscle strength and bladder control. Fabric-based dry electrodes (Figure 3) have been demonstrated for ECG [86], EEG [87], EMG [88] and FES [8]. At present all associated signal processing and control aspects are performed off the textile which increases the number of electrical connections required, reduces portability and ease of use. Further research is required to: (1) Improve dry electrode/skin contact and reduce resistance without using gel/water. (2) Incorporate the electrodes into user friendly garments and reduce the requirement for such tight-fitting clothing. (3) Improve the reliable integration of electronics in textiles.

### 4.3. Textile Pulse Oximetry

This technique is used in non-invasive monitoring of oxygen saturation of a patient’s blood. Conventional fingertip or earlobe-based pulse oximetry sensors are common place. Polymer fibre optics embroidered into a textile [89] and fabric-embedded LEDs and photodiodes [90] have demonstrated the feasibility of textile implementations. Neither approach has been developed into a practical solution. Further work is required to ensure robust and reliable operation and studies are required to validate performance.

### 4.4. Strain Gauges

Strain gauges, including piezoresistive (measure strain through a change in resistance) or capacitive (change in capacitance) are used in activity monitoring and classification or monitoring breathing by measuring strain or bending in a textile/garment. Existing solutions include screen printed resistive material with sewn conductive thread contacts [91], knitted resistive sensors [92,93] and multilayer capacitive assembly using conductive textiles and dielectric layers [94]. Further work required on cyclical stability of sensors and minimising drift, sensor placement vs. activity type, activity classification and sensor fusion (combining data with output from other sensor types).

### 4.5. Temperature Sensors (e.g., Thermistor-Change in Resistance with Temperature)

This type of device is used in general health monitoring and feedback control in heated garment applications (e.g., improving circulation). Discrete temperature sensors have been embedded into textiles and commercialised products are available on the market (e.g., Siren smart socks for diabetic foot temperature monitoring). Off-the-shelf thermistors have been soldered onto a copper wire and encapsulated to form a resin pod which was integrated into textiles [95]. Bare die thermistors have been integrated with complete circuits in the FETT project utilizing flexible circuit fabrication techniques and bespoke packaging [96]. Applications specific challenges remain and the influence of the different thermal properties of textile fibres and structures on the response of embedded temperature sensors requires evaluation.

### 4.6. Moisture Sensors

These are used in incontinence applications. Disposable urine activated batteries can be used to provide a self-powered system detecting urinations [97]. Carbon nanotube-based filament sensors that change resistance with moisture can be fabricated as a yarn [98]. Existing lab-based demos need to be developed for the application, cost reducing and manufacturing challenges remain, sensor robustness and reliability need to be established.

### 4.7. Textile Pressure Sensors

**Figure 4:**
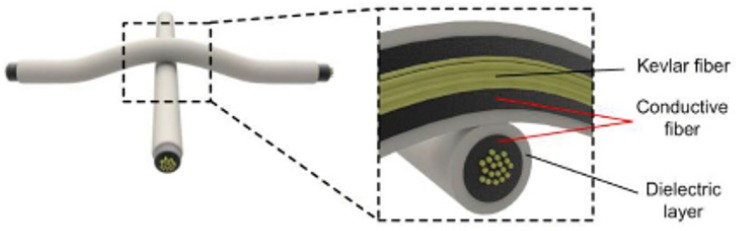
Pressure sensitive resistive fiber based on Kevlar fibers coated with a silver nanoparticles and surrounded by a dielectric rubber material [99].

**Figure 5:**
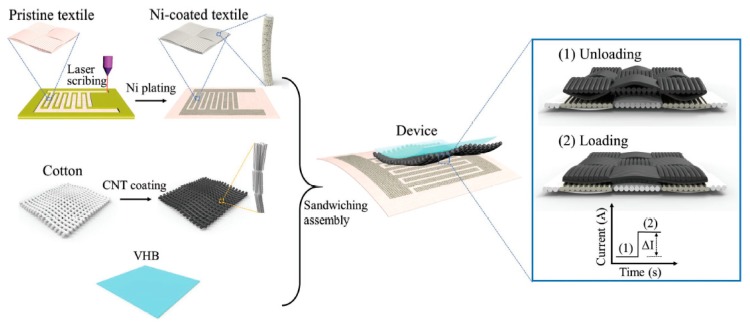
The fabrication of textile pressure sensor based upon nickel (Ni) and carbon nano tube coated textiles [101].

Such sensors are used in managing bedsores, foot-based balance monitoring for fall detection and prevention, and compression garments. Resistive based approaches including dielectric coated conductive yarns (Figure 4) have demonstrated good performance in terms of sensitivity and stability [99]. A similar concept has been commercialised by Texis [100]. Other sensor types have been demonstrated in the lab (e.g., carbon nano tube coated fabrics shown in Figure 5 [101] and piezoresistive textiles [102]). Remaining challenges include improving stability and longevity, scaling up for large area production and ensuring washability.

### 4.8. Fabric-Based Glucose Sensors

These devices are used to manage glucose levels include discrete monitors for flash and implanted sensors and new sweat based glucose sensors. Electronics for remote monitoring of flash and implanted sensors can be integrated and hidden in clothing. Flexible circuits and packaging technology developed in FETT project is applicable [96]—this requires development and testing in the intended environment. Sweat based glucose sensors would be well suited to a textile platform but whilst the feasibility of using sweat has been demonstrated [65], a fabric-based glucose sensor has yet to be demonstrated. Research into textile compatible electrochemical sensor for glucose monitoring and associated electronics, packaging and validation is required.

### 4.9. Actuators

Actuators are used in assisting mobility and providing haptic feedback. Fibre-based twisted and coiled soft actuators based on Spandex can contract by 45% when resistively heated to 130 °C [103]. Electrostatic actuation demonstrated with woven soft PVC fabric sandwiched between two electrodes suitable for generating vibrations [104]. Pneumatic actuators (artificial muscles) have been demonstrated in a power-assist suit [105] and orthotic device [106]. Pneumatic exoskeleton suits are commercially available but these are not integrated in a textile. Research challenges include overcoming the high-power consumption of thermal actuators, formulating materials and soft robotic structures that maximise strain whilst minimising energy consumption. There is a requirement to develop soft fibres, yarns and fabric structures that maximise the strain achieved in electroactive actuators. Pneumatic actuation is capable of high forces but is poorly integrated into textiles and bulky. Research is required to improve integration, reduce size and engineer actuators and garments such that forces are coupled effectively to the wearer.

### 4.10. Fabric Speakers

These can enable audible communication with users for memory prompts and other assisting technologies as well as playing music. Active materials have been used to generate sound from textiles. A ferroelectret actuator (thin foam-based material driven by applied voltage) has been sandwiched between fabrics to make a music playing flag [107]. Piezoelectric materials such as PVDF have also been used [108] to enhance the sound pressure. Current driven printed conductive spirals on textile have been combined with a permanent magnet to generate sound [109]. Research is required to increase the actuating force of the active materials whilst retaining compatibility with fabrics, improve coupling between the actuating materials and the textile and investigate the influence of garment design on speaker performance.

### 4.11. Breathing Sensors

Breathing sensors are used in measuring sleep quality, assisting with breathing exercises and improving technique, and detecting speech (social interaction). Breathing rate and volume can be determined by a number of methods, for example, measuring local strain resistive sensors on the skin [110] and on textiles [111]. Strain sensitive fibre optic sensors have been glued to a textile [112]. Other novel sensing techniques demonstrate include RF frequency [113] and capacitance (e.g., capaciflector [114]). Inertial sensors can be used [115], but have yet to be demonstrated implemented in a textile. The fabric-based sensing techniques demonstrated are all in the early stages of development and require further work to improve integration and achieve reliable operation over a long-term basis. Performance also needs to be benchmarked against existing systems.

### 4.12. Integrated Location Sensors

These are used in identifying an individual’s location and movements both within the home and outside. Rigid electronics modules that use a combination of GPS and inertial sensors to track the performance of elite athletes are commercially available. These are typically inserted into a pocket in the clothing at the top of the back [116]. Research is required to improve the integration of the technology into the clothing, such that it is invisible both to the user and externally. Textile GPS antennas have been widely researched but no textile-based GPS/inertial system has been demonstrated. Flexible electronics and packaging approaches such as that developed in the FETT project need to be applied in this application and benchmarked.

### 4.13. Antibacterial Textiles

Textiles with antibacterial properties are used in smart bandages. Antimicrobial fabrics and associated finishing products are commercialised and used, for example, in medical environments [117] and bandages. Research challenges remain in improving the environmental friendliness of passive fabrics and preventing the migration of silver nanoparticles that reduces efficacy. Wound treatment can be improved with using smart bandages that include active functionality such as sensing and controlled drug release [118]. Ultraviolet (UV) light in the correct wavelength can actively kill bacteria, however incorporating UV emitting sources into a bandage has not yet been demonstrated.

### 4.14. Textiles for Releasing Drugs

Textile-based drug releasing products have been demonstrated using a variety of techniques. Passive approaches contain active ingredients in, for example, the form of microscopic capsules that break under mechanical strain or abrasion. Such approaches provide an uncontrolled release mechanism but active approaches activated using, for example, embedded heaters can release the drug as required. Trapped biomolecules released in response to a specific thermal stimulus have been demonstrated in an active dressing for chronic wounds healing [119]. Research is required to characterise and improve the response of textile fibres and drug infused coatings to a range of stimuli and all such methods will require proper clinical evaluation.

### 4.15. Heated Fabrics

Finally, these are used to improve circulation and aid recovery from certain injuries. Heated clothing is already widely commercialised based upon resistive heating (for example Blaze Wear [120]) and there are no significant fundamental research challenges remaining. There are potential challenges when considering specific applications such as thermally controlled drug release from fabrics [119].

For the technologies listed above, there are a wide range of major challenges required to advance the e-textile embodiments to a point at which they offer a practical solution and potentially a commercially viable product. In general, fundamental research is required to improve the sensitivity, repeatability, durability, reproducibility and level of integration of the textile-based technologies enabling their use in long-term monitoring and support systems. Manufacturing infrastructure is required to enable large scale production and sufficient evidence is need to validate users’ need and obtain regulatory approval for medical devices. This is aligned with the challenges identified in published review papers [121,122,123,124]. The research topics relating to these challenges can be grouped into the following categories:

(1) Materials and their performance in textiles—There have been a wide range of functional materials been used in healthcare care applications including fabric-based dry electrodes for monitoring, diagnosis and treatment; flexible stretchable resistive and piezoelectric materials for sensors; electroactive polymer for actuators (e.g., artificial muscles); carbon nanotube-based filament and dielectric coated conductive yarns for conductors and heaters; light emitting polymers for therapies. These materials must survive the rigours of use in the relevant application scenarios. Existing e-textiles are typically unsatisfactory in terms of reliability during use and durability (e.g., bending, stretching, washing). Biocompatibility (e.g., cytotoxicity, irritation, sensitisation) is also an essential requirement to ensure user safety and comfort is another issue.
(2) Discrete sensor/device integration in textiles—Electronics (e.g., inertial sensors, pulse oximetry, temperature, circuit) and textile integration has progressed in three generations [125]. First generation e-textiles attached conventional rigid electronics to textiles i.e., the textiles acted only as a platform for the electronics (e.g., Philip-Levi ICD jacket [126]). Second generation e-textiles embedded functional devices such as switches and sensors into the textile [127]. Third generation e-textiles integrate flexible electronic functionality, including circuits [96], at the yarn level in e-yarns with significantly reduced size thus allowing the potential for electronics to be unobtrusive and effectively hidden within the textile. However, challenges remain on e-yarn length, reliable interconnections, and component sizes and flexibility limit integration.
(3) Manufacturing—The widespread manufacturing of e-textiles has been limited by the diverse range of techniques required to produce a set of functions in an e-textile. Ideally the e-textiles manufacturing process should be compatible with existing equipment (e.g., spinning, knitting, weaving, printing, coating, dyeing, finishing) already in use for conventional textile manufacturing to enable mass production. This is not straightforward and the specific challenges will depend on the electronics functionality and manufacturing process. For example, printing functional materials requires a much higher quality print than would be the case for patterning a fabric since any errors will result in failure.
(4) Regulatory—Compliance with medical device regulations is necessary for medical devices in order to demonstrate the safety and clinical effectiveness. This has set an entry barrier and lengthened the time required to take medical devices to market. The change in the EU medical device regulation with the Medical Device Regulation (MDR) replacing the Medical Device Directive (MDD), with full effect from May 2020, will make the entry barrier even higher. Clinical evaluation (e.g., randomized controlled trials) are needed for new emerging products.
(5) End users need validation—The majority of scientific research has been carried out in the lab with little or no input from end users. This leads to a disconnection between the technology and the end user requirements which delays uptake and the development of new products. Involvement of the end user and other key stakeholders (e.g., clinicians, regulators) from the outset of the project is essential to ensure the research effectively addresses the users’ need and smooths the transfer from lab research to adoption in the market.

It should be noted that this review has focused on the research challenges that relate *in particular* to healthy ageing. It has deliberately not included more generic technical challenges that are common to e-textile applications in general. These more generic challenges include the supply of electrical power, wireless communications and associated textile antenna design. The amount of research in this area covering the topics presented in this review is increasing steadily with the number of publications on the topics of smart fabrics and e-textiles rising by 170% from 2015 to 2018. Over the next 10 years e-textiles should move increasingly out of the laboratory and into practical applications driven initially by military, safety/workwear and sports and fitness applications. Health related applications will certainly follow although these can take longer due to the requirements of regulatory approval.

## 5. Conclusions

It is clear that as the corresponding technologies continue to evolve, wearable healthcare systems based on e-textiles will be an attractive solution for comfortable and un-obtrusive monitoring of health parameters and providing treatment for ageing related diseases. Achieving the required functionality and performance and using scalable manufacturing approaches compatible with conventional textile methods such as weaving, knitting and printing have a great potential for developing low-cost wearable solutions.

Research is required to improve the sensitivity, repeatability, durability, reproducibility and level of integration of the textile-based technologies enabling their use in long-term monitoring and support systems. Research topics can be grouped into materials, discrete sensor/device integration in textiles, manufacturing, compliance with regulatory requirement, and validation of the end users’ need. In order to efficiently address these research challenges, the range of disciplines involved in e-textiles research should be broadened. The multidisciplinary nature of the research challenges is clearly evident and developing solutions requires a complementary blend of skills and expertise. At present e-textiles research is often approached by researchers with a background in textiles or electronics but there is clearly the opportunity to increase the involvement of other communities such as materials scientists, chemists, manufacturing and instrumentation engineers, and researchers in healthcare disciplines. The research challenges can only effectively be met by interdisciplinary teams sharing expertise and methods and involving end users and other stakeholders at an early stage in the research. This combination is necessary to appreciate the requirements of the application and constraints imposed by textile manufacturing methods and application environments.
